# Coinfection with 2 *Clostridium difficile* ribotypes in China: A case report

**DOI:** 10.1097/MD.0000000000009946

**Published:** 2018-03-30

**Authors:** Liqian Wang, Yun Luo, Chen Huang, Shenghai Wu, Julian Ye, Xiaojun Song, Dazhi Jin, Xianjun Wang

**Affiliations:** a Department of Laboratory Medicine, Zhejiang Chinese Medical University Affiliated Hangzhou First Hospital; b Department of Microbiology, Zhejiang Provincial Center for Disease Control and Prevention; c Department of Laboratory Medicine, Hangzhou First Hospital, Huansha road, Hangzhou, Zhejiang, China.

**Keywords:** *Clostridium difficile* infection, ribotype 017, severe symptoms

## Abstract

**Rationale:**

*Clostridium difficile* infections (CDIs) have been reported in China, but detailed clinical symptoms of coinfection by 2 *C difficile* ribotypes have not been documented.

**Patients concerns:**

An 83-year-old male with a 10-day history of diarrhea and urinary tract infection was admitted to the hospital. The patient had received ofloxacin for several days, but his clinical response was poor. Laboratory workup revealed high white blood cell (WBC), serum creatinine (Scr), and C-reactive protein (CRP) levels. Based on these abnormal lab results, rapid detection of glutamate dehydrogenase and toxin A and B was performed.

**Diagnosis:**

Severe CDI.

**Interventions:**

Oral vancomycin was administered for 8 days.

**Outcomes:**

Diarrhea symptoms improved and *C difficile* culture was negative after oral vancomycin administration for 8 days. *Clostridium difficile* was isolated from 3 consecutive stool samples at 2-day intervals because the patient was admitted to the hospital. Polymerase chain reaction ribotyping revealed ribotype (RT) 017 in the first 2 samples and RT 001 in the third sample. RT 017 caused significantly higher increases in the levels of WBC, Scr, and CRP than RT 001.

**Lessons:**

It is necessary to improve clinicians’ awareness of CDI and reduce the severity of CDI caused by RT 017 in China.

## 1 Introduction

*Clostridium difficile* is a gram-positive, spore-forming anaerobe that causes symptoms ranging from mild diarrhea to toxic megacolon, colonic perforation, and death.^[1]^ The hypervirulent ribotype (RT) 027 has emerged in Europe, North America, and Asia, and produces more TcdA and TcdB toxins than other ribotypes.^[2]^ Since 2003, *Clostridium difficile* infection (CDI) prevalence, severity, and fatality rates have increased dramatically.^[3]^ Although most toxigenic strains produce both TcdA and TcdB (A^+^B^+^) toxins, strains producing only TcdB (A^−^B^+^) have been reported. Some A^−^B^+^ strains have been associated with severe cases.^[4,5]^

The epidemiology of CDI has changed dramatically in recent years. Several pathogenic A^−^B^+^ strains have emerged in Asia and Latin America.^[5]^ Inconsistent with its clinical presentation in North America and Europe, several RT 027 cases in China were not associated with severe symptoms.^[6]^ However, severe CDI cases caused by RT 017 have been documented.^[5]^ In this study, we have documented, for the first time, a single patient successively infected by 2 different ribotypes in China, and demonstrate that RT 017 caused more severe symptoms than RT 001.

## 2 Case report

**Figure 1:**
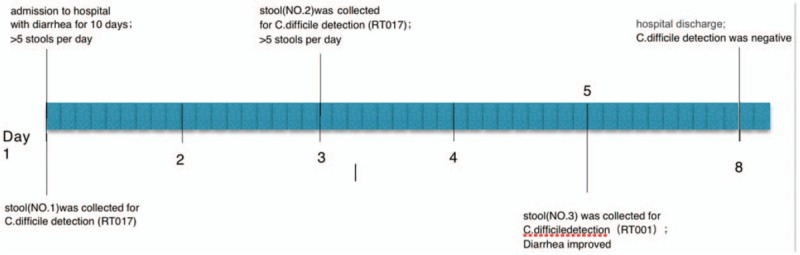
Course of *Clostridium difficile* infection in the patient.

An 83-year-old male patient with hypertension was admitted to the hospital owing to a 10-day history of diarrhea and urinary tract infection. He received ofloxacin for several days prior to arrival to the hospital. Laboratory workup revealed a white blood cell (WBC) count of 16.2 × 10^9^/L, serum creatinine (Scr) content of 52.7 g/L, and a C-reactive protein (CRP) level of 87 mg/L (Table 1). Based on these abnormal lab results, CDI was suspected. Rapid detection of glutamate dehydrogenase (GDH) and toxin A and B (C. diff Quik Chek Complete, Techlab, Blacksburg, VA) was performed using the patient's stool, which was positive for both GDH and the toxins. According to Society for Healthcare Epidemiology of America/Infectious Diseases Society of America guidelines,^[7]^ the case was categorized as severe CDI. Therefore, antimicrobial therapy was switched to oral vancomycin at a dose of 125 mg every 6 h. The course of the patient's disease is shown in Fig. 1. The study was approved by the Ethics Committee, and the informed consent was signed by the patient.

**Table 1:**
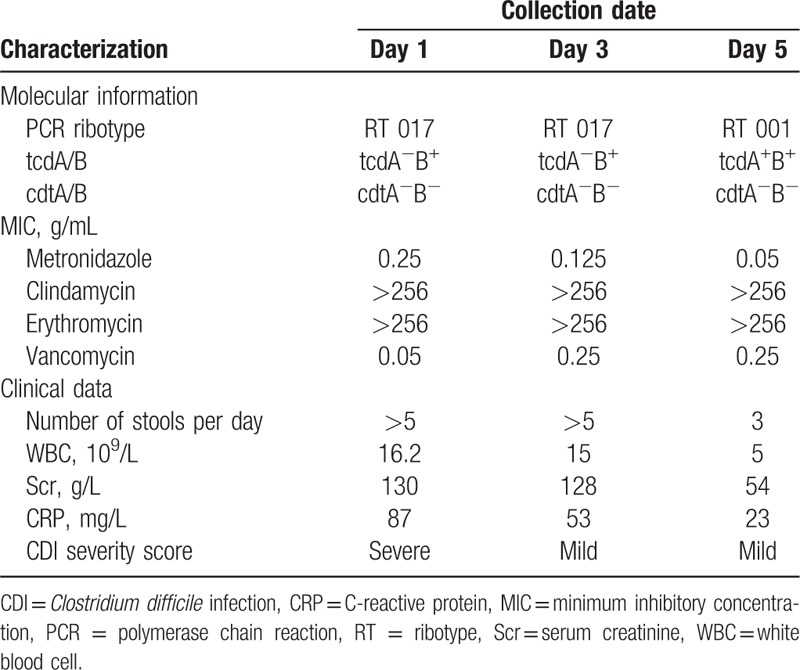
Information on the 3 isolates found in the patient.

Because the patient had severe diarrhea, we collected stool samples on the 1st, 3rd, and 5th days after hospitalization. All of the diarrhea samples tested positive for *C difficile* by matrix-assisted laser desorption ionization (Bruke, Karlsruhe, Germany), performed according to the manufacturer's instructions, with a cut-off score > 2.0. Bacterial genomic DNA was extracted using a QIAamp DNA Blood Mini Kit (Qiagen, Valencia, CA) according to the manufacturer's instructions. The housekeeping gene, toxin genes *tcdA* and *tcdB*, and binary toxin genes *cdtA* and *cdtB* were detected by previously described assays.^[8]^ Ribotyping was performed by polymerase chain reaction followed by capillary gel electrophoresis, as described previously,^[9]^ and as indicated in Table 1. The patient tested positive for RT 017 (day 1), RT 017 (day 3), and RT 001 (day 5). *Clostridium difficile* isolates were tested for susceptibility to metronidazole, vancomycin, clindamycin, and erythromycin, using E-test strips (AB Biodisk, Durham, NC) as previously reported.^[10]^ The antibiotic susceptibility of the 3 *C difficile* isolates is presented in Table 1. All isolates were resistant to clindamycin and erythromycin, but sensitive to vancomycin and metronidazole.

## 3 Discussion

CDI caused by BI/NAP1/RT 027 strains with both TcdA and TcdB (A^+^B^+^) toxins is estimated to cause an economic burden of $3.2 billion per year in the United States.^[11]^ Although the RT 027 strain was recently reported in China,^[6]^ RT 017 with only TcdB (A^−^B^+^) was one of the dominant ribotypes in 2 surveys of Chinese hospitals in Beijing^[12]^ and Hangzhou.^[13]^ The RT 017 strain is the main cause of moderate-to-severe CDI in hospitals in eastern China, as reported in our previous study.^[5]^ This case, with a single patient successively infected with 2 ribotypes, suggests that RT 017 causes more severe clinical symptoms than RT 001, as indicated by the number of stools per day, as well as increased WBC, Scr, and CRP levels (Table 1), which are commonly reported symptoms of CDI.^[14]^

Unique to this case, the patient was infected with 2 different ribotypes of *C difficile.* To rule out the presence of mixed ribotypes in stool samples, 100 suspected colonies were ribotyped in each of the 3 diarrhea samples, and only 1 ribotype was identified in each stool (Table 1). Therefore, we speculate that RT 001 might have come from the environment. However, because environmental sampling was delayed for a week and the ward had been repeatedly disinfected, we were not able to culture RT 001 from the hospital environment. Future environment sampling will be conducted in our hospital to aid in CDI epidemiology.

It is noteworthy that a patient with severe diarrhea and high-risk factors for CDI was not diagnosed until 10 days after the presentation of symptoms. CDI has not attracted sufficient attention in China, and a lack of clinical suspicion is the underlying reason for this issue. Thus, it is necessary to improve clinicians’ awareness of CDI and strengthen the cooperation between clinicians and clinical laboratories to detect and treat CDI in a timely manner. In summary, we report a continuous CDI with 2 different ribotypes, of which RT 017 caused more severe symptoms. This case provides the basis for a clinical follow-up study of CDI in China, and confirms that RT 017 is associated with severe clinical symptoms.

## Author contributions

**Conceptualization:** D. Jin, L. Wang, S. Wu, X. Wang, X. Song, Y. Luo.

**Data curation:** C. Huang, D. Jin, J. Ye, L. Wang, S. Wu, X. Wang, X. Song, Y. Luo.

**Formal analysis:** C. Huang, D. Jin, L. Wang, X. Wang, Y. Luo.

**Funding acquisition:** D. Jin, X. Wang.

**Investigation:** L. Wang, X. Wang.

**Methodology:** C. Huang, D. Jin, L. Wang, X. Song.

**Project administration:** D. Jin, L. Wang, Y. Luo.

**Resources:** J. Ye, L. Wang, S. Wu, X. Wang.

**Software:** C. Huang, S. Wu, X. Song, Y. Luo.

**Supervision:** D. Jin, X. Wang.

**Validation:** D. Jin, J. Ye, X. Wang.

**Visualization:** D. Jin, X. Wang.

**Writing – original draft:** L. Wang, X. Wang.

**Writing – review & editing:** D. Jin, L. Wang, X. Wang.
